# Present taxonomic work on Staphylinidae (Coleoptera) in Canada: progress against all odds

**DOI:** 10.3897/zookeys.186.3252

**Published:** 2012-04-26

**Authors:** Adam Brunke, Jan Klimaszewski, Robert S. Anderson

**Affiliations:** 1 Zoological Museum (Natural History Museum of Denmark), University of Copenhagen, 15 Universitetsparken, Copenhagen, Denmark, 2100 DK; 2 Natural Resources Canada, Canadian Forest Service, Laurentian Forestry Centre, 1055 du P.E.P.S., P.O. Box 10380, Stn. Sainte-Foy, Québec, Quebec, Canada G1V 4C7; 3 Research Division, Canadian Museum of Nature, P.O. Box 3443, Ottawa, Ontario, Canada K1P 6P4

The Staphylinidae or rove beetles represent one of the largest evolutionary radiations on earth with more than 56,000 species (Grebennikov and Newton 2009, Ślipiński et al. 2011) and are a dominant element of the insect fauna in many terrestrial ecosystems (examples discussed in Thayer 2005). Even though such an enormous diversity may seem unmanageable, we are living in an exciting time for taxonomic research on rove beetles. There has been a relatively sudden expansion in the field of bioinformatics, including e-taxonomy (see ZooKeys special issue 150 for further information). A world catalogue of Staphylinidae (minus Aleocharinae, Paederinae, and Pselaphinae) was recently published (Herman 2001) and followed by catalogues of the entire family for the Palaearctic (Löbl and Smetana 2004), and for the south temperate fauna (Newton and Thayer 2005). American Beetles Volume 1 (Newton et al. 2001) provided the first complete and modern keys to the genera of Staphylinidae occurring in North America north of Mexico, and Navarrete-Heredia et al. (2002) provided the same for Mexico. ‘The Beetles of Central Europe – Staphylinidae I’ by Assing and Schülke (2012) is the most complete species-level review and key for a regional staphylinid fauna to date, and represents a benchmark for other regions of the world to aspire to.

In the last quarter of the 20th century, Canada was a world leader in rove beetle systematics, providing two positions for full-time research (A. Smetana and J.M. Campbell) and one for technical support (A. Davies), in Biosystematics Research Centre, Agriculture Canada, Ottawa. Significant progress was made by these scientists in the study of Canadian staphylinid biodiversity, much of which was summarized in a catalogue of the Canadian fauna (Campbell and Davies 1991). Today, unfortunately, neither of these first two positions exists and financial support for biodiversity research in Canada and worldwide has shifted toward more interpretative studies that use a small fraction of biodiversity and open doors to higher impact journals. Fortunately, taxonomic research of the Canadian staphylinid fauna continues, albeit in a diminished and dispersed capacity.

Despite this unfavourable climate for descriptive taxonomy in Canada, the demand for baseline taxonomic data, basic information on the distribution, abundance and habitat preferences of species, continues. The routine implementation of biodiversity inventories (e.g. ATBI’s, and BioBlitz’s) has become important to conservation authorities, who need baseline data to make informed conservation decisions about the properties under their stewardship (e.g. OMNR 2009). Most manuscripts included in this special issue on Biodiversity of Canadian Staphylinidae include these data collected as a result of partnerships between taxonomists and governments (e.g. Ontario Ministry of Natural Resources, New Brunswick Department of Natural Resources (Fish and Wildlife Branch) New Brunswick Museum, and Canadian Wildlife Service) or non-profit organizations (e.g. Nature Conservancy of Canada and Meduxnekeag River Association). Baseline data included in this special volume improve our knowledge of biodiversity in imperilled Canadian ecosystems such as the Canadian Arctic (Yukon) and highly fragmented old-growth forests (New Brunswick and Ontario). The sustainable management of Canada’s rich natural resources also relies on baseline data to establish comparisons between reference (unaltered) and managed ecosystems under various degrees of resource extraction (e.g. Dollin et al. 2008, for Staphylinidae in managed forests). Baseline data are also used by ecologists in the approximation of ecological conditions using indicator species (Büchs 2003) and in modelling studies (Canhos et al. 2004). Additionally, a poor understanding of species distributions in a country as large and diverse as Canada severely limits studies of local and global biogeography. Researchers aiming to answer these questions depend greatly on an accessible and robust taxonomic knowledge base for their focal taxon and, given its fragmented state for Canadian Staphylinidae, this highly diverse and ecologically dominant group may be largely unavailable to the scientific community.

**Figure 1.**
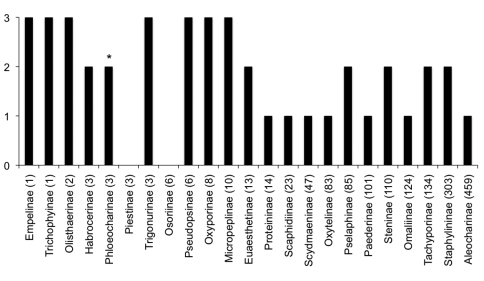
Level of taxonomic maturity and approximate number of species in Canadian Staphylinidae subfamilies: ‘0’ denotes mostly unrevised subfamilies; ‘1’ denotes maturing subfamilies with some modern revisionary works; ‘2’ denotes nearly mature subfamilies with modern revisions for most or all genera; ‘3’ denotes mature subfamilies post modern revision, with reviews and keys available in some format. *Phloeocharinae is ranked here as nearly mature because current keys do not separate the recently introduced *Phloeocharis subtilissima* Mannerheim from all native members of the subfamily.

Currently, taxonomic knowledge of Staphylinidae in Canada (and generally in North America) remains inadequate, especially when compared with the level of understanding attained for the Central European fauna. To appreciate the incompleteness of this knowledge and provide direction for future research, 23 subfamilies of Staphylinidae occurring in Canada were ranked according to the taxonomic maturity of the majority of their genera (Fig. 1), with a minimum of ‘0’ representing mostly unrevised groups and a maximum of ‘3’ representing a level comparable to that in Central Europe: post-revision, with a review and checklist of taxa, and with keys available in some form. Of 23 subfamilies, only 7 can be considered well known and accessible (ranked ‘3’), and all are species-poor in Canada or in general. Of the remaining 16, 10 subfamilies are speciose in Canada but only 4 of these can be considered nearly mature, with modern revisions available for most genera. This is due primarily to modern descriptive work by D.S. Chandler and J.A. Wagner (Pselaphinae), A. Smetana (Staphylininae), V. Puthz (Steninae) and J.M. Campbell (Tachyporinae). References to most of these important works are included in the “literature cited” sections of the articles featured herein. Subfamilies with poorly understood biodiversity in Canada are: Aleocharinae, Omaliinae, Osoriinae, Oxytelinae, Paederinae, Piestinae (*Siagonium*), Proteininae (*Proteinus*), Scaphidiinae and Scydmaeninae; we recognize however, that several studies on these groups are currently in progress. Notably, the situation in the subfamily Aleocharinae is steadily improving with monographs by Gusarov (e.g. 2003, 2004) and Génier (1989), and more recent collaborations between the second author (JK), and R. Webster (New Brunswick), C. Majka (Nova Scotia and the Maritime Provinces), D. Langor (Newfoundland and Alberta), B. Godin (Yukon Territory), and N. Winchester (British Columbia).

The present contributions to this special issue on the Biodiversity of Canadian Staphylinidae improve our knowledge of these poorly known subfamilies and provide valuable baseline data about their taxonomy, distribution, collection methods and habitat preferences. In this issue, there are three contributions to the biodiversity of Canadian Aleocharinae (Webster et al. 2012, Brunke et al. 2012, and Klimaszewski et al. 2012), and collaborative investigations by R. Webster and others on Omaliinae, Osoriinae, Oxytelinae, Paederinae, Piestinae and Scaphidiinae. Even in subfamilies that are better known in Canada, the present contributions to the knowledge of Habrocerinae, Micropeplinae, Olisthaerinae, Oxyporinae, Phloeocharinae, Pselaphinae, Staphylininae and Tachyporinae clearly demonstrate that much remains to be discovered and documented concerning Canada’s rich biodiversity heritage. This special issue is published in the spirit of this exploration and for all individuals with a passion for rove beetle biodiversity.
